# Supervised learning for the automated transcription of spacer classification from spoligotype films

**DOI:** 10.1186/1471-2105-10-248

**Published:** 2009-08-12

**Authors:** David J Jeffries, Neil Abernethy, Bouke C de Jong

**Affiliations:** 1 MRC Laboratories, Fajara, The Gambia; 2 Department of Medical Education and Biomedical Informatics, University of Washington, USA; 3 Division of Infectious Diseases and Immunology, New York University, USA

## Abstract

**Background:**

Molecular genotyping of bacteria has revolutionized the study of tuberculosis epidemiology, yet these established laboratory techniques typically require subjective and laborious interpretation by trained professionals. In the context of a Tuberculosis Case Contact study in The Gambia we used a reverse hybridization laboratory assay called spoligotype analysis. To facilitate processing of spoligotype images we have developed tools and algorithms to automate the classification and transcription of these data directly to a database while allowing for manual editing.

**Results:**

Features extracted from each of the 1849 spots on a spoligo film were classified using two supervised learning algorithms. A graphical user interface allows manual editing of the classification, before export to a database. The application was tested on ten films of differing quality and the results of the best classifier were compared to expert manual classification, giving a median correct classification rate of 98.1% (inter quartile range: 97.1% to 99.2%), with an automated processing time of less than 1 minute per film.

**Conclusion:**

The software implementation offers considerable time savings over manual processing whilst allowing expert editing of the automated classification. The automatic upload of the classification to a database reduces the chances of transcription errors.

## Background

Genotyping of *M. tuberculosis *complex isolates has enhanced TB control and contact tracing while providing valuable insights on tuberculosis transmission and pathogenesis [1]. Recently, strain differences were found to affect clinical presentation [2], and unravelling of the genes responsible for these phenotypic differences might lead to the identification of drug- and vaccine targets.

Spacer oligonucleotide typing (spoligotype) analysis is the most user-friendly and commonly applied genotyping tool for *M. tuberculosis *isolates worldwide. Global spoligotype databases include 'fingerprints' from thousands of *M. tuberculosis *complex isolates from diverse regions [3]. Based on hybridization of the direct repeat region of *M. tuberculosis *[4], spoligotype analysis generates reproducible binary patterns of 43 spacers, which can readily be shared electronically. This 43 binary spacer format can be transcribed as a 15-digit code [5], although no international standardization has been established. While spoligotype analysis lacks the resolution of the 'gold standard' genotyping method IS*6110 *restriction fragment length polymorphism (RFLP) [6], it has several advantages compared with this technique: Firstly, it relies on PCR amplification of *M. tuberculosis *DNA, which requires much less DNA and can be applied straight to sputum samples. Secondly, up to 43 isolates can be completed within one day. Thirdly, isolates with less than 6 bands on IS*6110 *RFLP can be genotyped with a higher resolution by spoligotype analysis. Finally, the spoligotype patterns can distinguish between subspecies and clades within the *M. tuberculosis *complex and are phylogenetically informative [7].

Spoligotyping generates arrays of spots and typically data entry and classification is performed manually, which can result in errors [8], or with often expensive electrophoresis gel-type software. Spoligotype analysis is a robust method with reproducibility of over 90% [9], but it can be affected by the subjective determination of the hybridization signal, especially after repeated use of the membrane. Hybridization detection is not an all-or-nothing process and slight variations in the quality and repeatability of results requires these images to be double checked by experienced staff. Alternatively a fully automated multiplex bead-based Luminex hybridization assay can be used [10]. Whereas several papers have described novel data mining methods for spoligotype data [11,12], to our knowledge none have developed software to facilitate the acquisition of the images and classification of spots on a complete spoligotype film generated by membrane based hybridization. Commercial software packages have facilities for the semi-automated capture and classification of spoligo film images. The BioNumerics platform has a 'Character Types' module which can process spot information from tif files. Quantity One (Bio-Rad) allows images to be segmented to quantify spot information via the 'Volume Tools' function.

To support the spoligotype analysis of a Tuberculosis Case Contact study in the Gambia [13], we used two supervised learning algorithms, a neural network [14] and a support vector machine [15] to categorize the hybridization signal as positive or negative. These algorithms were incorporated into a software package that automated the process of gridding, classification, expert verification and transcription of data from the scanned spoligo film directly into a database.

## Results

Spoligotype analysis was performed according to a standardized method [4] using commercially prepared membranes from Isogen Biosciences (Maarsen, the Netherlands). The assay was repeated for isolates with spacers that were difficult to classify, and for those without any hybridization signal.

The spoligotype film was then scanned using a transparency enabled scanner (Microtek ScanMaker i800) at a low resolution of 300 pixels per inch, with a 256 level greyscale. Transparency enabled scanners have a light source in the lid of the scanner as well as the base. The images were scanned to tiff files (although any common image file type can be used), appropriately cropped and rotated so that the rows (isolates) were true horizontal. The automated processing of spoligo films can be divided into four main processes, automatic grid placement, feature extraction, supervised learning and data storage.

### Algorithm – Automatic grid placement

**Figure 1:**
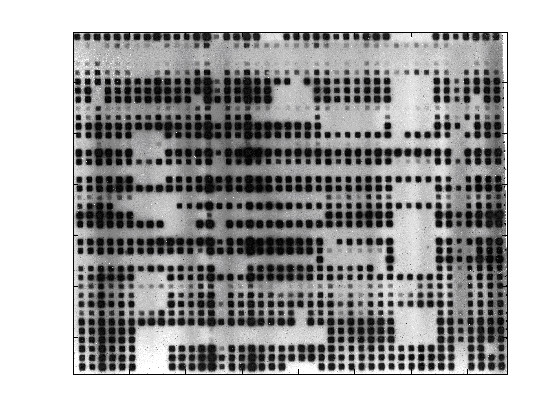
**Typical spoligo film with non-orthogonal rows and columns, and blank and smudged areas**.

**Figure 2:**
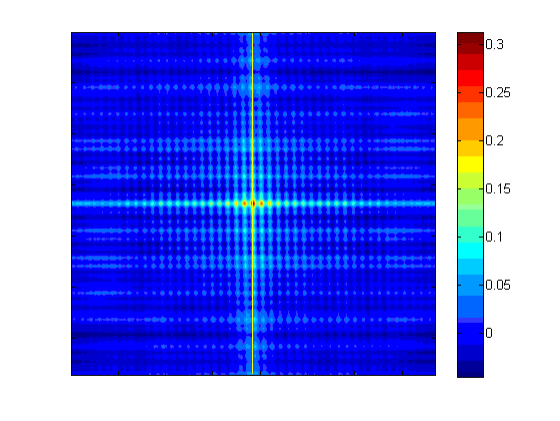
**Heat map of auto-correlation of spoligo film with non-orthogonal rows and columns**.

**Figure 3:**
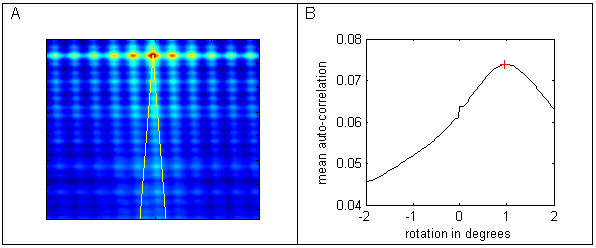
**Extracting auto-correlations**. Figure 3A heat map of film autocorrelation, with arcs of ± 2° from film centre. Figure 3B mean auto-correlation along each arc.

There are two key problems with automatic gridding. Firstly, due to the physical process of developing films the rows and columns are not necessarily orthogonal. Secondly, the spoligotype films have small irregularities in the width of the spacer lanes and isolate rows, often with blank or smudged areas. To address these irregularities, we used a 2 dimensional autocorrelation of the spoligo image to identify non-orthogonal rows and columns. For an M by N image matrix x, the autocorrelation requires the calculation of:



where i = -M to M and j = -N to N and elements of x that lie outside its range are set to zero. Image sizes tend to be approximately 1500 by 1500 pixels, which make the above summation computationally expensive. The autocorrelation is calculated by taking the inverse Fourier transform of the product of the Fourier transform of the image and its complex conjugate (Wiener-Khinchin Theorem). Figure 1 shows the image of a spoligo film with non-orthogonal rows and columns. The non-othogonality can be seen in the image autocorrelation shown in Figure 2, where the columns are not aligned with the true vertical yellow line. The angle from the true vertical for the columns can be calculated by finding the arc through the centre with the highest correlation. Figure 3A shows an arc between ± 2 degrees and Figure 3B gives the mean intensity sweeping in 101 steps from -2 to + 2 degrees, where the highest correlation occurs at an angle of 0.96 degrees. To calculate the mean intensity along an arc, a 'nearest neighbour' 2 dimensional interpolation is used as the arcs do not pass exactly through a pixel.

The image could now be sheared to make the rows and columns orthogonal, but this creates an edge effect problem and the displayed image is no longer identical to the film. For these reasons the grid lines were rotated rather than the image.

The number of columns in a film is set at 43 and the number of rows has a maximum of 43. Given these parameters it is trivial to place an initial grid, allowing for the rotation affect. Each grid line is then moved -15 to +15 pixels (0 to 15 for edges) from its initial position and the mean intensity for each position is calculated. White areas in the image have high intensity values and dark areas low values and the grid line is moved to the area of highest intensity.

**Figure 4:**
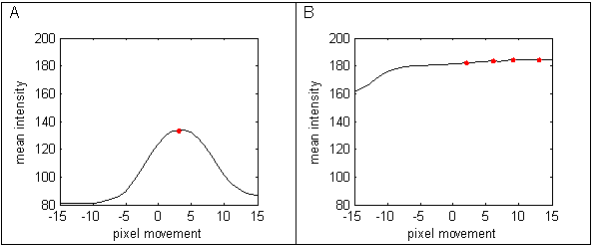
**Row mean intensity profiles for perturbation of horizontal grid lines**. Figure 4A shows the profile for moving a grid line between rows with many dark cells. Figure 4B shows the profile moving the grid line through noisy light areas, with few dark cells.



The films typically have rows missing due to failed isolates or controls and these areas must be detected as noise because they have very few low intensity dark pixels. Figure 4A shows the profile for the grid line between two well defined rows in comparison to Figure 4B where the second row has very few defined cells. A bump hunting algorithm is used to locate the local maxima and the following threshold is calculated, where c is the number of maxima and m_i _are the mean intensities for each perturbed grid line.

For noisy data with no well defined dark cells the numerator tends to be large and the denominator small, and for the example in Figures 4A and 4B, T_noise _= 0.11 and 3.93 respectively. Based on trial and error the threshold was set at 0.93, which successfully relocated the initial grid lines for the 874 lines on the 10 films (note one film only had 38 isolates) analysed here, using the following criterion.

If c>0 and T_noise _< 0.93

Move grid line to the highest intensity peak

Else

Do not move grid line

**Figure 5:**
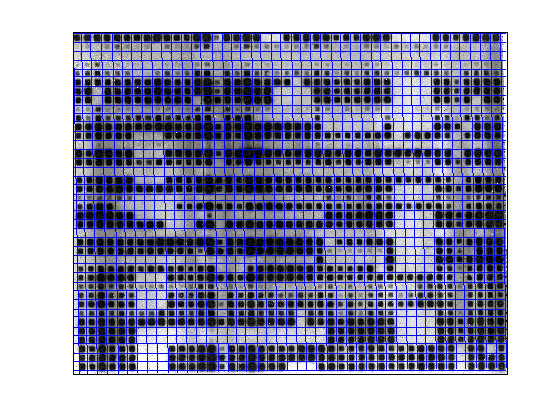
**Automatic grid placement, where rows and columns are non-orthogonal**.

After the optimum position for a grid line was determined the remaining lines were moved to maintain the initial separation and the process is repeated for each grid line in turn. Figure 5 shows the automatic grid lines for the film in Figure 1 and individual cells are defined by the four intersections of the relevant rows and columns. The automated software implementation allows any line to be manually moved, but the classification process was not sensitive to small movements in the grid lines.

### Algorithm – Feature extraction and supervised learning

The success of any supervised learning technique depends on the ability of the features extracted to discriminate. There is no prescriptive rule for the determination of these features and the selection difficulty is compounded in this case by the wide range of film quality and the large number of cells that have to be processed. Features that discriminate on low noise/high quality films might not be the same as those on films of inferior quality. Given that the automated implementation has to run in real time, a small number of features should be extracted from each cell. Initially simple summary statistics are extracted from each cell, i.e. the mean and the median pixel intensity for each cell.

The largest dark region within a cell is then selected, as follows. The intensity of the pixels is thresholded, by setting those below the lower quartile intensity for the cell to white and the remaining pixels to black. The largest of the resulting black regions is selected as the region of interest. The vertical and horizontal profiles of the cell pixels through the centre of this region are then extracted and smoothed. The profiles are smoothed using a B-splines smoother [16], with a uniformly high degree of smoothing, to reduce the number of noisy peaks. The B-splines smoother is appropriate for a real time application as it has a fast computational implementation. The amplitude of each smoothed profile and the number of minima are calculated. Positive cells typically yield profiles having well defined centrally located minima and large amplitudes, in contrast to negative cells.

**Figure 6:**
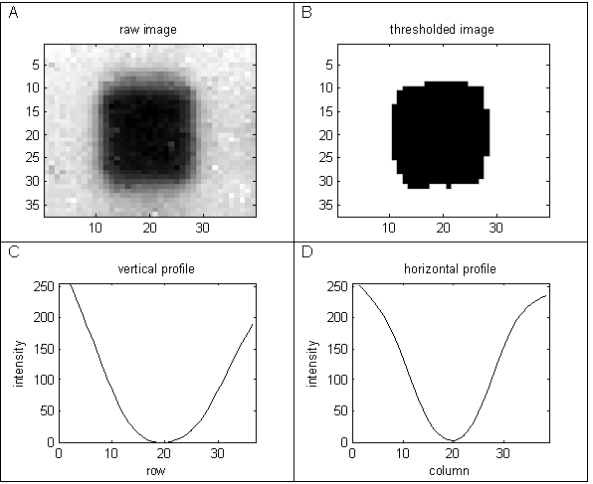
**Feature extraction from a positive cell with a central feature**.

**Figure 7:**
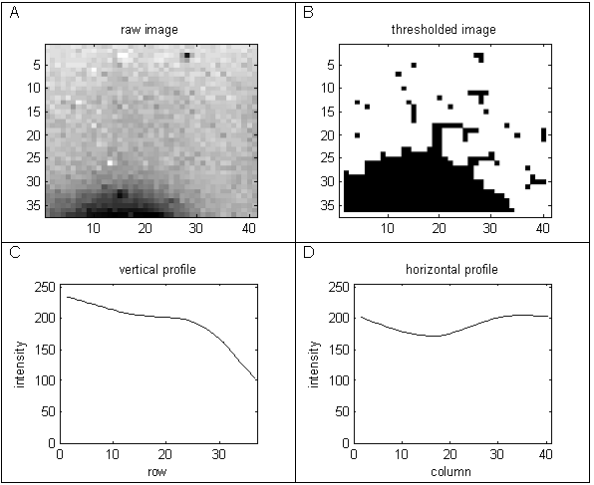
**Feature extraction from a negative cell with an edge artefact form a neighbouring cell**.

Due to the non-uniformity of the spot sizes, there can be edge effects caused by overlap from neighbouring cells. These cells can be identified using a symmetry index, calculated by taking the largest central square image from a cell and rotating it by 90 degrees and calculating the correlation coefficient of the pixel intensities with the un-rotated square image. Cells with large central features tend to have higher correlation than cells with noise or non-symmetrical edge features. Figures 6 and 7 compare the horizontal and vertical profiles for a positive cell with a central spot to those from a negative cell having an edge artefact. For these cells the most effective discriminator is the rotational symmetry coefficient, which is 0.84 for the image in Figure 6 and -0.13 for the image in Figure 7.

This gives seven features which are fast to compute (computations have to be repeated for up to 43*43 cells) and are able to discriminate between positive and negative cells from a range of different quality films. At the expense of information, the dimensionality of multivariate training sets can be reduced using principal component analysis [17]. For this application where the dimensionality of the features is small and a wide range of film qualities may have to be processed, there is little computational advantage in attempting to reduce the dimensionality.

Based on a training set of the seven extracted features where the true classification of the cell is known, supervised learning algorithms can classify cells based on unseen sets of cell features. The implementation of two common supervised learning algorithms, a neural network and a support vector machine are described here and compared in the following section.

A feed forward neural network links inputs to outputs by a series of one way connections. The inputs are the seven features for each cell and there are two outputs representing a positive or negative cell. A unary encoding is used for the outputs in the form of a 2 element vector [a b], where a perfect prediction is [0 1] and [1 0] for a positive and negative cell respectively.

Between the input and output layers there is at least one set of neurons, which connects the inputs to the relevant outputs for the training set. More complex relationships can be modelled by increasing the number of neurons and/or increasing the number of layers between the inputs and outputs. There is no prescriptive method for determining the number of neurons or hidden layers and for the films classified here, 2 layers with 5 neurons in each layer gave good performance across all films. The number of layers and neurons are parameters that can be easily changed. The network was trained using back-propagation and the Levenberg-Marquardt algorithm was used to optimise the weights. One of the key problems with neural networks is overfitting, where the error on the training set is very small, but the network does not generalise well when used to process unseen data. To overcome this problem the training sets are divided into two, a training and a validation set. The validation set is classified at each iteration (epoch) during training, based on the weights obtained from the training set only. If the network over fits the data, the error on the validation set tends to increase. An early stopping rule is applied if this occurs for 6 consecutive iterations and the network based on the minimum of the validation error is returned. The requirement for the neural network to converge in a real time implementation restricts the size of the training set. Simulation showed little improvement in classification performance for sets of more than 3% of the cells, split evenly between the training and the validation set.

Support vector machines are closely related to neural networks. They construct a hyperplane separating the input vectors into two categories and so are ideal for binary classification problems. Support vector machines have integral support to minimize overfitting by creating a soft margin between categories that allows some misclassifications. To allow non-linear separations a kernel function is used to transform the data and in contrast to determining the numbers of neurons and hidden layers for a neural network, only the appropriate kernel needs to be chosen for a support vector machine. Simulation showed that a linear kernel function gave the most appropriate fit, with non-linear fits showing overfitting problems. In common with the neural networks simulation, a training set of 1.5% of the cells was adequate. Given the integral support for overfitting and the use of a linear kernel no further control for overfitting was necessary and a validation set was not required.

A neural network is initialized with a set of random weights and for repeat training sessions it will converge to different classifications from the same training set. This is in contrast to a support vector machine, which results in a one to one relationship between the training set and the classification.

### Algorithm – Data storage and retrieval

The data from the classified films are automatically updated to an Access data base, although any common database standard such as SQL server or Oracle could be substituted. Each film is stored as a separate table, where each row is labelled with a unique isolate identifier and a row position identifier. To enhance data quality and portability, film images are stored directly within the database (see implementation for technical details).

### Testing

The algorithms above were tested on ten films, where quality ranged from very high, to images with uneven exposure and noisy artefacts. The gold standard for comparison with the supervised learning algorithms was provided by manual classification by an experienced laboratory technician and verification by a senior scientist. Visual confirmation by the above staff showed good automatic grid placement for the ten films and a neural network and a support vector machine were applied to each spoligotype film, using identical training sets.

**Figure 8:**
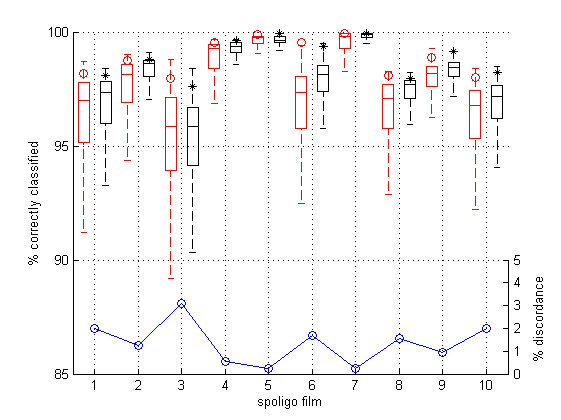
**Comparison of neural network and support vector classifiers**. Symbols compare the percentage of correct classifications from 10-fold cross-validation for a neural network (red) and support vector machine (black). Box plots compare the classifiers based on 1000 training sets from each spoligo film and the blue line shows the median percentage discordance.

The two classifiers were compared using 10-fold cross-validation. For a real time practical implementation, the training sets of 1664 cells generated by10-fold cross-validation on a complete film of 1849 spots are not feasible. To test the dependence of the classification on realistic training sets, 1000 randomly selected training sets (the only constraint was that they must contain both positive and negative cells) consisting of 1.5% of the cells (with a validation set of 1.5% of cells for the neural network), were generated. Figure 8 shows (neural network red o, support vector machine black *) the percentage of correctly identified cells by cross-validation. The box plots in Figure 8 summarize the distribution of correct classifications from the 1000 simulations of smaller training sets for each film. The reduction in the accuracy of the classifiers when using small training sets is greatest in the poorer quality films. The median percentage discordance between the two classifiers for each film is shown by the blue line in Figure 8.

A rank based analysis of variance on the percentage of correct classifications, allowing for the clustering effect of film [18], shows no significant difference (p = 0.90) between the classifiers for the 10-fold cross-validation. For the data simulated from smaller training sets the support vector machine showed a border line significant improvement (p = 0.047) over the neural network. The smaller training sets compared to those from the 10-fold cross-validation resulted in a significant (p < 0.001) performance degradation for both classifiers. Generally for realistic training sets the support vector machine gives slightly better classification, shows less dependence on the training set and is computationally faster to fit. In terms of real time software implementation for small training sets there is little functional difference between the two methods.

The best films have median correct classification rates above 99%. For the poorer quality films there is as expected a lower classification rate and greater dependence on the training set selection. The results in Figure 8 are conservative as the training sets were chosen randomly and there was no constraint to include training cells having diverse characteristics. In reality decisions about difficult to classify cells may depend on neighbourhood cells and exposure artefacts, which is why the software implementation facilitates user re-classification. The worst performance occurred for the third film, which has a very uneven spot exposure making classification difficult even by manual inspection.

**Figure 9:**
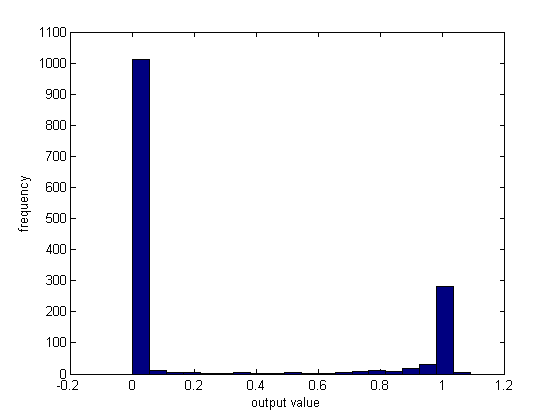
**Histogram of first element from neural network classifier output**.

The outputs from either a neural network or a support vector machine can be used to highlight the quality of cell classifications. The output from the neural network is a vector with 2 rows and the same number of columns as cells classified. Each column is an estimate of the classification, where [0 1] and [1 0] are perfect positive and negative predictions and well classified cells are polarized in these directions. The output from a support vector machine is the distance from the hyperplane and the sign of the distance determines the category of a cell. Figure 9 shows the histogram of the first row of the output vector from a neural network for the third film, representing the poorest classification. There are clearly some values around 0.5, indicating poor classification. Cells within this region can be tagged as likely to be poorly classified. In a similar manner for a support vector machine the distances from the hyperplane clustered around 0 could be used to identify difficult to classify cells.

There are many different image processing morphological operations and filters that attempt to improve the quality of image, but these had little affect on the classification error for the poorer quality films. The disadvantage of image transformations is that the film image can be noticeably different from the actual film, which doesn't promote user confidence. The only transformation that was universally applied and accepted was a simple contrast stretch.

### Implementation

Software for the preceding applications was developed in Matlab (The Mathworks Inc version R2008b), which offers a rich program development environment and dedicated image processing and neural network toolboxes. Matlab is compatible with MAC OSX, Linux and Windows operating systems. Although it is ideal for developing and testing code, the language is interpreted, which makes some of the more intensive numerical operations relatively slow, affecting the user-friendliness of this interactive software application. Code written and compiled in C++, can be incorporated into Matlab applications, resulting in faster execution speed, often greater than 10 fold. The Fast Fourier transform, two-dimensional interpolation, and the morphological operations are written in C++.

The code is controlled from a graphical user interface and films are defined by their image name, where the actual image can be in any of the common graphical file types. The code then runs (on a machine with a Xeon 3.4 GHz CPU and 1 GB of RAM) in the following order (user actions in italics):

1. User selects film for processing from file browser

2. Automatic grid placement (5 seconds)

3. User can manually move grid lines if required

4. Extracts features from all cells (15 seconds)

5. User chooses training set and marks any regions excluded from classification

6. User chooses classifier (Neural network or support vector machine)

7. Trains and displays classified film (1 second)

8. User verifies and edits classification

9. Writes classification and film image to a Microsoft Access database (1 second)

Even though the process is completely automatic, the user can override any of the computer generated results. Every line of the grid is a selectable graphics object and the lines can be moved forward, backwards, up or down one pixel at a time. After classification any of the cell classifications can be over-written before updating the results to a database. Connection to the Access database is via an object linking and embedding database connector and all database actions are automatically written from the Matlab code using SQL (structured query language), so no knowledge of Access is required. Rather than storing a link to the spoligotype film image; for integrity and database portability the actual film image is stored directly in the database. This requires the image to be converted to a Blob (binary large object) and written from Matlab to an OLE (object linking and embedding) field in the relevant database table. A typical resolution spoligotype film after resizing by a factor of a quarter consumes 500 kB of space in the database table. Storage within a single Access database is only limited by the product limit of 2 GB, which would not affect a server sided database such as an SQL server.

**Figure 10:**
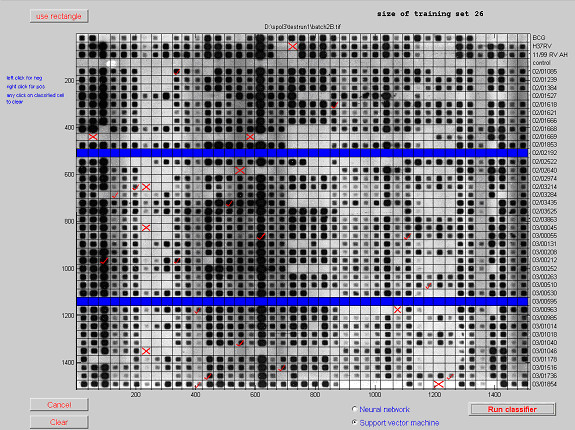
**Definition of training set and marking of failed isolates (rows in blue)**.

**Figure 11:**
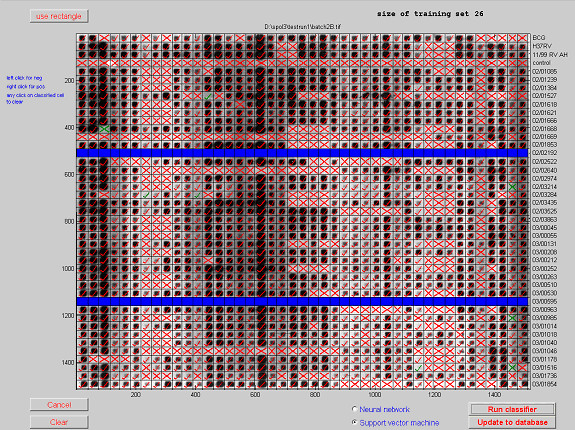
**Support vector machine classification**.

Figure 10 shows the graphical user interface for defining the training sets, with a user entered training set of 1.5% of cells for a support vector classification. Rectangular regions or individual cells can be classified by the user and as cells are added the displayed size of the training set is incremented. Failed isolates can be marked in the same manner (shown in blue) and are then excluded from the classification process. Either of the classification techniques discussed in this paper can then be implemented. The results from the chosen classifier (a support vector machine in this case) are then displayed directly onto the film as shown in Figure 11. The red crosses or ticks are the output from the selected classifier and can be re-classified by the user, by right clicking on any cell. The classifications shown in green are those cells with the weakest classification. After verification and re-classification the results are automatically up-loaded to the database, which occurs instantaneously. The unique isolate identifiers shown to the right of the film can be extracted from any convenient source such as a database, Excel or a text file and are then stored with the data in the database.

The results in Figure 8 were obtained using randomly selected training sets, but intelligent choice of the training set is likely to give superior performance. The film in Figure 11 is film 1 from Figure 8 and had a median correct classification of 97.4%. The support vector machine classification displayed in Figure 11 correctly classified 98.1% cells with 8 cells having weak classification (marked with green ticks and crosses), including 3 incorrectly classified cells.

Films can be re-classified at any time and users are warned before updating any existing classifications in the database. An additional graphical tool displays previously classified films and allows cell classifications to be viewed and if necessary edited.

## Discussion

We developed a method for automating the transcription of spacer classification from spoligo films. With a large throughput of films from the laboratory, the process had to offer a high degree of automation, but allow manual re-classification and provide a systematic method for storing the data. There appear to be no commercial packages exclusively for the processing of spoligo films.

Matlab is a high level programming language with excellent graphical capabilities, dedicated image processing, supervised learning tools and sophisticated native support for database connectivity, which make it ideal for processing and managing spoligo film data. C++ can also be incorporated into Matlab programs, allowing this application to run within a realistic timeframe.

After scanning, the processing of the spoligofilms was divided into four categories, grid application, classification, manual verification and data storage. The automated processes run in less than 1 minute, which is a considerably less than manual processing. The implementation described here uses either a neural network or a support vector machine as classification tools. They provide robust classification, with the support vector machine showing superior performance on realistically sized training sets. In terms of the number of cells correctly identified it is of little practical relevance. Given the speed of film specific classifiers there is little advantage in using the same global classifier for multiple films. There are many other types of classification algorithm, which could easily be incorporated into the modular code.

The Matlab source code is available for download from the 'statistics and data management' page of The Gambia MRC website  and from the corresponding author. It requires the image processing, neural network and bioinformatics (for support vector machine) toolboxes. Bespoke code or alternative classifiers could be used to replace the reliance on toolboxes. Data is currently output to a Microsoft Access 2007 database, but there is native support for all the database systems most commonly used in medical research. The modular nature of the code makes it straightforward to add additional output formats. For example the input format for the spolTools software [19] is text with format "name: binary pattern: cluster size" (where cluster size refers to the number of isolates with a particular pattern). The Matlab code stores output data in a two-dimensional matrix which can easily be written to a text file, from where it could be pasted into the spolTools page. Although this paper has concentrated on films with 43 spacers, the only limitation on the addition of further spacers [8] is the size of the scanner.

Many aspects of the gridding, feature extraction, and supervised learning are generally applicable to other laboratory image-based analyses, and could be adapted to the analysis of microarray or well-plate images. Further research will optimise the software implementation, explore the development of platform independent executable code and compare the speed and accuracy of traditional manual data entry against classification with the automated method.

## Conclusion

We developed a user friendly software package that can capture and classify data generated by reverse hybridization methods, such as spoligotype analysis. Although fully automated the software allows for manual editing, before uploading the data to an Access database. Tools are also provided to visualize existing data and make retrospective changes. The software is publicly available, and potential users can contact us for assistance, modification and additions to the software.

## Availability and requirements

The Matlab (version R2000b) source code, user guide and anonymous test film images are available from the 'statistics and data management' page of The Gambia MRC website  and the corresponding author. Note the source code requires the image processing, neural network and bioinformatics toolboxes.

## Authors' contributions

DJJ developed the algorithms and the Matlab code. NA researched image processing algorithms and advised on the application. BCDJ highlighted the issues with manual processing and extensively tested the application. All three authors contributed to the writing of the paper.
